# Neuroinflammation and Neurometabolomic Profiling in Fentanyl Overdose Mouse Model Treated with Novel β-Lactam, MC-100093, and Ceftriaxone

**DOI:** 10.3390/toxics12080604

**Published:** 2024-08-19

**Authors:** Mohammed S. Alasmari, Fawaz Alasmari, Shakir D. Alsharari, Abdullah F. Alasmari, Nemat Ali, Syed Rizwan Ahamad, Abdullah M. Alghamdi, Aban A. Kadi, Alaa M. Hammad, Yousif S. Mohamed Ali, Wayne E. Childers, Magid Abou-Gharbia, Youssef Sari

**Affiliations:** 1 Department of Pharmacology and Toxicology, College of Pharmacy, King Saud University, Riyadh 11451, Saudi Arabiaffalasmari@ksu.edu.sa (F.A.); sdalsharari@ksu.edu.sa (S.D.A.); afalasmari@ksu.edu.sa (A.F.A.); nali1@ksu.edu.sa (N.A.); 437101495@student.ksu.edu.sa (A.M.A.); 437103561@student.ksu.edu.sa (A.A.K.);; 2 Department of Pharmaceutical Chemistry, College of Pharmacy, King Saud University, Riyadh 11451, Saudi Arabia; srahamad@ksu.edu.sa; 3 Department of Pharmacy, College of Pharmacy, Al-Zaytoonah University of Jordan, Amman 11733, Jordan; alaa.hammad@zuj.edu.jo; 4 Department of Pharmaceutical Sciences, Temple University School of Pharmacy, Philadelphia, PA 19140, USA; wayne.childers@temple.edu (W.E.C.); aboumag@temple.edu (M.A.-G.); 5 Department of Pharmacology and Experimental Therapeutics, College of Pharmacy and Pharmaceutical Sciences, University of Toledo, Toledo, OH 43606, USA

**Keywords:** fentanyl overdose, metabolomics, β-lactams, GLT-1

## Abstract

Opioid-related deaths are attributed to overdoses, and fentanyl overdose has been on the rise in many parts of the world, including the USA. Glutamate transporter 1 (GLT-1) has been identified as a therapeutic target in several preclinical models of substance use disorders, and β-lactams effectively enhance its expression and function. In the current study, we characterized the metabolomic profile of the nucleus accumbens (NAc) in fentanyl-overdose mouse models, and we evaluated the protective effects of the functional enhancement of GLT-1 using β-lactams, ceftriaxone, and MC-100093. BALB/c mice were divided into four groups: control, fentanyl, fentanyl/ceftriaxone, and fentanyl/MC-100093. While the control group was intraperitoneally (i.p.) injected with normal saline simultaneously with other groups, all fentanyl groups were i.p. injected with 1 mg/kg of fentanyl as an overdose after habituation with four repetitive non-consecutive moderate doses (0.05 mg/kg) of fentanyl for a period of seven days. MC-100093 (50 mg/kg) and ceftriaxone (200 mg/kg) were i.p. injected from days 5 to 9. Gas chromatography–mass spectrometry (GC-MS) was used for metabolomics, and Western blotting was performed to determine the expression of target proteins. Y-maze spontaneous alternation performance and the open field activity monitoring system were used to measure behavioral manifestations. Fentanyl overdose altered the abundance of about 30 metabolites, reduced the expression of GLT-1, and induced the expression of inflammatory mediators IL-6 and TLR-4 in the NAc. MC-100093 and ceftriaxone attenuated the effects of fentanyl-induced downregulation of GLT-1 and upregulation of IL-6; however, only ceftriaxone attenuated fentanyl-induced upregulation of TRL4 expression. Both of the β-lactams attenuated the effects of fentanyl overdose on locomotor activities but did not induce significant changes in the overall metabolomic profile. Our findings revealed that the exposure to a high dose of fentanyl causes alterations in key metabolic pathways in the NAc. Pretreatment with ceftriaxone and MC-100093 normalized fentanyl-induced downregulation of GLT-1 expression with subsequent attenuation of neuroinflammation as well as the hyperactivity, indicating that β-lactams may be promising drugs for treating fentanyl use disorder.

## 1. Introduction

Mortality rates from drug overdoses are a cause for concern and often indicate a public health crisis [1]. According to the Center for Disease Control and Prevention, the number of deaths from high doses of drugs has been estimated to be more than one million since 1999 [2]. Opioids, mainly fentanyl and its derivatives, are the main contributors to overdose related deaths with more than 70% of opioid overdose deaths are being attributed to synthetic opioids, primarily, fentanyl [3]. Therefore, it is of upmost importance to conduct further studies on different toxicological aspects of fentanyl overdose to provide new insights into the way that this health crisis could be managed. It is worth noting that fentanyl is a highly potent agonist at μ-opioid receptors (MOR) and has been indicated for the management of acute and chronic pain; however, recreational use may lead to overdoses, which can be fatal [4].

In the nucleus accumbens (NAc), the brain region responsible for reward and drug dependence, exposure to fentanyl overdose can cause mitochondrial dysfunction and oxidative stress [5], which may be explained by dysregulation of the glutamatergic system [6]. Glutamate is a very important excitatory neurotransmitter and significantly involved in several neurological functions, including learning, memory, and synaptic plasticity. For optimal brain function, it is essential for glutamate to be kept within the proper physiological levels to avoid excitotoxity and neuronal damage that may develop from excess of extracellular glutamate. Glutamate can lead to excitotoxicity; an effect considered as an important factor that might be involved in withdrawal and relapse to drugs of abuse [7]. Furthermore, dysregulation in glutamate homeostasis contributes to the development of opioid dependence as reported in preclinical and clinical studies [7,8,9,10]. The astrocytic glutamate transporter 1 (GLT-1) regulates the majority of extracellular glutamate concentrations in the brain [11] and is potentially considered as a target for the treatment of neurological diseases and psychiatric disorders involving hyperglutamatergic states [7,12,13,14]. Functional enhancement of GLT-1 in the brain may reduce the excitotoxic effects of the hyperglutamatergic state associated with abused drugs, including opioids.

β-lactams have been used in preclinical studies as well as clinical studies to upregulate the expression of GLT-1 or at least to attenuate the downregulation of this transporter that might be caused by exposure to drugs of abuse or diseases associated with hyperglutamatergic states [7,12]. Studies from our laboratories and others revealed the potential therapeutic effect of targeting GLT-1 to reduce glutamate excitatory signaling as well as neuroprotection with several β-lactams, including ceftriaxone [7,13,15]. Recently, MC-100093, a novel synthetic monocyclic β-lactam lacking antibacterial properties, was recognized as a potent GLT-1 upregulator, and it attenuated alcohol intake and cocaine seeking behavior [16,17,18].

Metabolomics is one of the omics techniques that have gained widespread adoption, and its utilization has broadened to encompass diverse scientific disciplines [19]. Metabolomic technology plays a central role in toxicological research by identifying metabolic response to drug toxicity, by understanding the underlying mechanism of toxicity, and by discovering druggable targets that can be modulated pharmacologically. It generates a comprehensive profile on cellular metabolites such as fatty acids, amino acids, and sugars, and it helps researchers gain new insights into metabolic pathways and a disease mechanism. In pharmacological and toxicological fields, this technique has been widely used to elucidate the mechanism of toxicity and to identify biomarkers that can be targeted therapeutically.

This study is an extension from a previous study, in which we investigated the effect of fentanyl overdose on the liver of mice treated with β-lactams [20]. However, in the current study, we focused on the NAc, which is significantly involved in the reward and drug dependence [21,22]. The effect of fentanyl overdose was evaluated in terms of metabolomic profile and the expression of GLT-1 and neuroinflammatory signaling, including interleukin 6 (IL-6) and Toll-like receptor (TLR-4). In addition, we evaluated the neuroprotective effects of β-lactams, ceftriaxone, and MC-100093 as modulators for the expression and function of GLT-1

## 2. Materials and Methods

### 2.1. Study Design

This study was approved by the Institutional Animal Care and Use Committee (IACUC) of the Research Ethics Committee in King Saud University with ethics reference number of KSU-SE-22-48. We used male BALB/c mice at the age of seven weeks. Prior to the start of the experiment, the mice were placed in cages, surrounded by identical suitable environmental conditions in a dedicated room for the preservation of experimental animals, provided with free access to food and water, and exposed to a 12 h light/dark cycle. The standard practice for handling experimental animals was followed to minimize stressful situations that may disrupt brain metabolic physiology. Animals were randomly allocated to one of the four experimental groups. The 1st group of mice was given a daily injection of intraperitoneal (i.p.) normal saline for 9 days, serving as a control group. The 2nd, the 3rd, and the 4th groups were given 0.05 mg/kg (i.p.) fentanyl every other day on days 1, 3, 5, and 7, and the dose of fentanyl was increased to 1 mg/kg as an overdose on day 9. It is important to note that from days 5 to 9, the 1st and 2nd groups received i.p. injections of saline vehicle, and the groups 3 and 4 received i.p. injections of ceftriaxone (200 mg/kg i.p.) and MC-100093 (50 mg/kg, i.p.), respectively. The mice were sacrificed on day 10, and the brains were immediately collected and stored at −80 °C. The NAc was micropunched from each brain group using a cryostat maintained at a temperature of −20 °C and stored for further metabolomic profiling and Western blot analysis.

### 2.2. Metabolomics Analysis

Untargeted metabolomic analysis was performed as described previously [20]. The analytical steps for metabolomics consisted of extraction of metabolites using ethanol, derivatization using gas chromatography–mass spectrometry (GC-MS), and metabolic profiling [23,24]. Briefly, under controlled environmental conditions, frozen samples were defrosted, weighed, and homogenized in methanol to extract the metabolites, and then centrifuged for 5 min at 10,000 rpm at 4 °C. After centrifugation, we transferred 200 μL of the supernatant into a 2 mL GC-MS vial. The samples were then dried using nitrogen. We further added 100 μL of 20 mg/mL methoxamine hydrochloride to the pyridine solution, followed by 10 min of vortex mixing and 16 h of incubation. After the incubation period, we derivatized the samples using BSTFA/TMCS (99/1, *v*/*v*), and injected 1 μL using a split-mode injection device (split ratio of 1:20). GC-MS was conducted using a PerkinElmer Clarus 600 gas chromatograph, a Clarus 600 T mass spectrometer, and Turbomass software. The MetaboAnalyst web-based platform was used for the analysis and interpretation of metabolomic data acquired from GC-MS. A set of analytical methods, including sparse Partial Least Squares Discriminant Analysis (sPLS-DA), heatmap analysis, scores plot, and enrichment analysis, has been supported by the MetaboAnalyst and was used to identify significant differences and patterns in the data.

### 2.3. Behavioral Tests

We comprehensively assessed locomotor activity in mice through the open field activity monitoring system for the evaluation of fentanyl-induced locomotive impairment. The mice were habituated for 10 min, then placed in open field boxes and allowed to explore for 10 min. During this period, the distance of animal movement was detected using activity monitoring software. Moreover, Y-maze spontaneous alternation performance (SAP) assay was used to assess spatial working memory and exploratory behavior. The Y-maze consists of three arms labeled A, B, and C, forming a Y shape. A mouse was initially placed in one arm (e.g., arm A) and allowed to freely explore the maze. The mouse was allowed to explore the maze for 10 min, and the sequence of entries into the arms was recorded. Spontaneous alternation was calculated as the percentage of entries in which the mouse chooses a different arm than its last two choices. For calculating SAP, we used the following formula: SAP = (# of triads with alteration/total # of triads −2) × 100. ‘Triads’ refer to sets of three consecutive arm choices. A higher percentage of spontaneous alternation is generally considered indicative of intact spatial working memory and exploratory behavior. A lower percentage may suggest deficits in memory or attention.

### 2.4. Western Blot Assay

The NAc was homogenized in 50 μL RIPA lysis buffer that was already mixed with proteolytic enzymes inhibitors. The homogenates were incubated for an hour on ice and then centrifuged, and the supernatants containing extracted proteins were further investigated. A Direct Detect^®^ spectrometer (EMD Millipore, Burlington, MA, USA) was used to quantify the extracted protein lysates. After mixing with 2× Laemmli buffer (Bio-Rad, Hercules, CA, USA), equal amounts of protein lysates were loaded into the wells of SDS-polyacrylamide gel electrophoresis (PAGE) for separation process. The voltage was set at 40–50 V for 5 min, and then was increased to 100–150 V for 1 h. For the transferring phase, we applied an electrical current in the presence of transfer buffer (25 mM Tris, 190 mM glycine, and 20% methanol) to transfer proteins into polyvinylidene difluoride (PVDF) membrane. Thereafter, the membrane was blocked for an hour using buffer that consists of 3% non-fat dry milk in a Tris buffered saline (TBS) solution, and an overnight incubation was conducted (at a temperature of 4 °C) with primary antibodies against the proteins of interest. The primary antibodies are rabbit anti-GLT-1, rabbit anti-IL-6, rabbit anti-TLR-4, and rabbit anti-β-actin. After incubation with the primary antibodies, the PVDF membrane was incubated with secondary diluted antibodies in 3% non-fat milk in 1 TBST for 1 h. We used a chemiluminescence-detecting reagent to detect band signals, ChemiDocTM MP imaging system (BIO-RAD) for visualization, and Image J software for the quantification of protein expression.

### 2.5. Statistical Analysis

Two-way analysis of variance (ANOVA) was used to analyze the average body weight between groups at baseline, during the repeated treatments exposure and after overdose phases. One-way ANOVA followed by Tukey’s post hoc analysis was used to analyze behavioral, metabolomic, and protein expression data, and to evaluate differences between groups. Data were presented as mean and standard error of the mean (SEM), and the *p*-value was set at <0.05 for significance. Network analysis and enrichment analysis represent the effect of fentanyl overdose group alone on the metabolic profile.

## 3. Results

### 3.1. Effects of Fentanyl Overdose and Beta-Lactams on Average Body Weight

**Figure 1:**
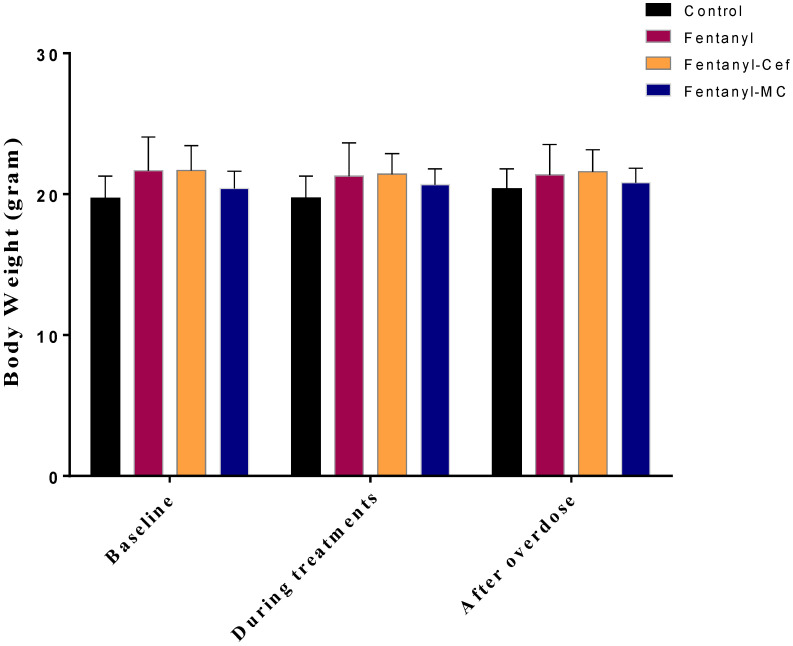
BWE Average body weight measurements of the control and experimental groups over time. Body weights were measured in the control, fentanyl, fentanyl/ceftriaxone, and fentanyl/MC-100093 groups at three time points: baseline (day 0), during treatment (day 5), and post treatment (day 9). Two-way ANOVA indicated no significant differences in body weight between groups at baseline, during treatment, and post treatment (n = 7/group). Cef; ceftriaxone, MC; MC-100093.

The average body weights of animals were monitored at baseline, during treatment, and at the end of the experiment. A Two-way ANOVA test showed no significant difference between the groups in all settings, for the time F (2, 50) = 1.641, treatment F (3, 25) = 1.642, and interaction F (6, 50) = 1.449 (Figure 1).

### 3.2. Effects of Fentanyl Overdose and Beta-Lactams on Locomotor Activity and Spontaneous Alteration Performance

**Figure 2:**
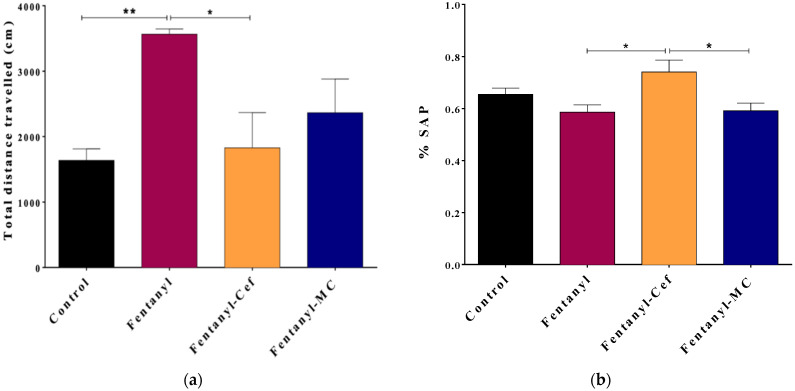
Results of the open field (**a**) and Y-maze (**b**) behavioral tests to assess locomotion and spatial working memory, respectively. Panel (**a**) depicts the total distance traveled by mice in 10 min period as an indication of locomotor activity. The fentanyl-alone group showed high locomotor activities compared to the control group. The fentanyl/ceftriaxone and fentanyl/MC-100093 groups showed locomotor activities comparable to the control group. Panel (**b**) shows the percentage of spontaneous alteration performance (SAP) as an indication of spatial working memory. There was no significant decrease in SAP within the fentanyl-alone group compared to the control group. The data are presented as mean ± SEM. (* *p* < 0.05, ** *p* < 0.01, n = 7/group).

To evaluate the effect of fentanyl overdose on the motor function, an open field test was conducted to measure the cumulative distance covered by the mice, as an indicator of locomotor activity (Figure 2A). One-way ANOVA test showed a significant difference in the total distance traveled between the control group and the fentanyl-alone group with mean ± SEM of 1638 ± 174 cm versus 3566 ± 79 cm, respectively, and the *p*-value of 0.007. Importantly, fentanyl-induced hyperlocomotion was attenuated significantly with ceftriaxone (1829 ± 539 cm), and to less extent with MC-100093 (2366 ± 515) as compared to the control group (Figure 2A).

Additionally, a Y-maze test was conducted to measure SAP for assessment of spatial working memory and exploratory behavior (Figure 2B). In comparison to the control group with the mean ± SEM of 0.6544 ± 0.024, the change in the percentage of SAP was not significant in fentanyl alone, fentanyl/ceftriaxone, and fentanyl/MC-100093 groups with the mean ± SEM of 0.586 ± 0.028, 0.741 ± 0.046, and 0.591 ± 0.0293, respectively (Figure 2B). Interestingly, the fentanyl/ceftriaxone group showed a significant improvement in SAP compared to the other fentanyl overdose groups, but this was not the case in comparison with the control group.

### 3.3. Effects of Fentanyl Overdose and Beta-Lactams on Metabolomic Profiling

**Figure 3:**
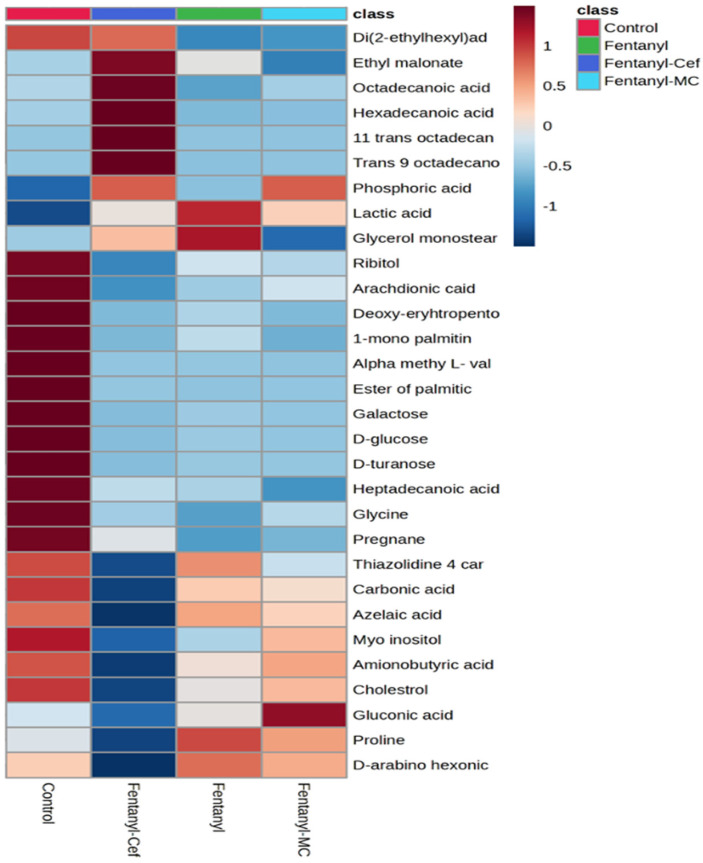
Heatmap displaying the relative levels of detected metabolites in the control group, fentanyl group, fentanyl/ceftriaxone group, and fentanyl/MC-100093 group. Each row represents a specific metabolite, and each column corresponds to experimental groups. The color scale ranges from blue (indicating lower metabolite levels) to red (indicating higher metabolite levels). The control group shows a baseline metabolite profile, while the fentanyl group exhibits significant alterations in metabolite levels compared to the control. The fentanyl/ceftriaxone and fentanyl/MC-100093 groups show a slightly different pattern of metabolite changes, suggesting a minimum modulatory effect of β-lactams on fentanyl-induced metabolic alterations (n = 5–7/group).

Data derived from the analysis of metabolites were visualized using heatmap to identify the overall patterns in the abundance of the NAc metabolites as a result of fentanyl overdose (Figure 3). In the heatmap matrix, rows correspond to significantly altered metabolites, and columns represent different experimental groups. A color scale has been applied to represent the intensity of metabolite abundance. Typically, a gradient from low to high abundance is used, with dark blue indicating a very low abundance, while dark red indicating a high abundance of metabolites. The heatmap analysis showed an overall alteration in the NAc metabolomic profiling of several metabolites as a result of fentanyl overdose. We have identified substantial differences in the abundance of several key metabolites between the control and fentanyl groups. For instance, ribitol, arachidonic acid, glycine, D-glucose, D-turanose, and pregnane were decreased with fentanyl overdose. These altered metabolites were not modulated in the fentanyl/MC-100093 and fentanyl/ceftriaxone groups. However, in comparison with the fentanyl-treated group, diethylhexyl adipate and glycerol monostearate metabolites were modulated in the fentanyl/ceftriaxone and fentanyl/MC-100093 groups, respectively. Overall, the heatmap analysis revealed distinct metabolic signatures associated with fentanyl overdose, suggesting potential biomarkers of interest for further investigation.

We assessed the changes in the levels of metabolites between the control and fentanyl groups using the peak area ratio. The peak area ratio for each metabolite was determined through (1) identifying the peak area of the metabolite, (2) calculating the average peak area of the metabolite in the control group, and (3) dividing the metabolite’s peak area by the average peak area of the control group. This process was repeated for each metabolite to determine its ratio relative to the control group. About 30 metabolites in the NAc, including carbohydrates, amino acids, and fatty acids, exhibited significant differential peak ratios between the control and fentanyl overdose groups.

**Figure 4:**
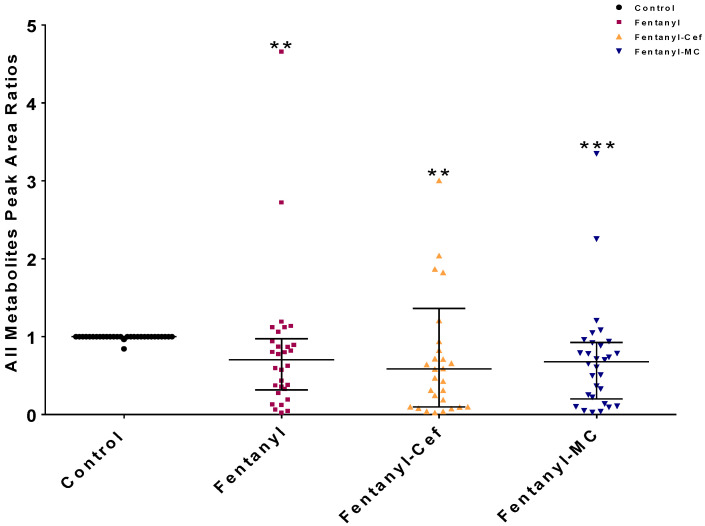
One-way ANOVA showed significant changes in the metabolomic profiles of groups exposed to fentanyl overdose compared to the control group. Each dot in the graph represents the mean of one metabolite in each group. The data are reported as a mean of all metabolites means ± SEM. (** *p* < 0.01, *** *p* < 0.0001, n = 5–7/group). Cef, ceftriaxone; MC, MC-100093.

We statistically confirmed the significant metabolic alteration as a result of fentanyl overdose using one-way ANOVA statistical analysis (Figure 4). According to the overall peak area ratios for disturbed metabolites, one-way ANOVA statistical analysis confirmed the significance of the observed differences between the control group and all fentanyl overdose groups (*p* < 0.01) as shown in Figure 4. However, there was no significant difference between fentanyl- and both fentanyl-β lactams-treated groups. The significant decrease in the overall peak area ratio in the fentanyl groups suggests that the fentanyl overdose led to a broad reduction in metabolites levels.

**Figure 5:**
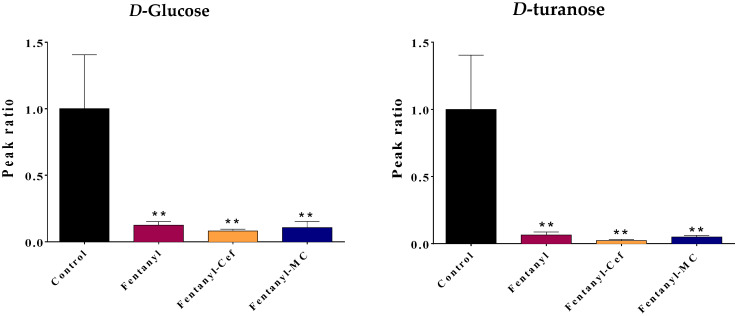
Effect of fentanyl overdose with and without β-lactams on the level of carbohydrates glucose and turanose. The bar graph illustrates the mean ratio of the metabolite peak area (normalized to the control group) across the four groups: control, fentanyl overdose, fentanyl overdose with ceftriaxone, and fentanyl overdose with MC-100093. The error bar represents SEM. The control group is set as the baseline (ratio = 1), while the fentanyl-alone group exhibits a significant decrease in the metabolite ratio. No significant difference was found between the fentanyl and fentanyl/β-lactams groups (** *p* < 0.01, n = 5–7/group). FEN: fentanyl, CEF: ceftriaxone, MC: MC-100093.

**Figure 6:**
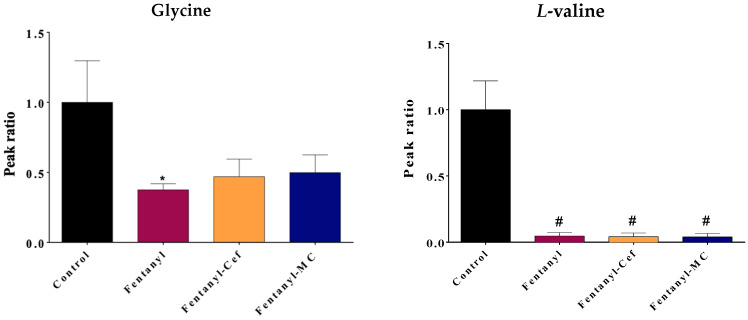
Effect of fentanyl overdose with and without β-lactams on the level of amino acids glycine and valine. The bar graph illustrates the mean ratio of the metabolite peak area (normalized to the control group) across the four groups, and the error bar represent SEM. The control group is set as the baseline (ratio = 1), while the fentanyl overdose group exhibits a significant decrease in the metabolite ratio (* *p* < 0.05, # *p* < 0.0001, n = 5–7/group). FEN: fentanyl, CEF: ceftriaxone, MC: MC-100093.

**Figure 7:**
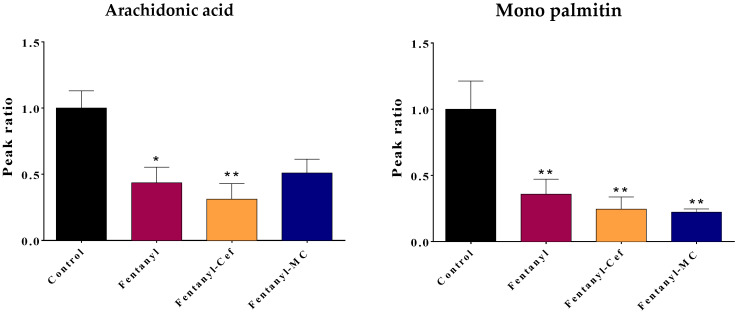
Effect of fentanyl overdose with and without β-lactams on the level of arachidonic acid and mono-palmitin. The bar graph illustrates the mean ratio of the metabolite peak area (normalized to the control group) across the four groups, and the error bar represent SEM. The control group is set as the baseline (ratio = 1), while the fentanyl overdose group exhibits a decrease in the metabolite ratio (* *p* < 0.05, ** *p* < 0.01, n = 5–7/group). FEN: fentanyl, CEF: ceftriaxone, MC: MC-100093.

Some metabolites were selected for graphical representation based on their potential to provide valuable insights into the underlying mechanism and strength of statistical significance (Figure 5, Figure 6 and Figure 7). The selected metabolites are known to play crucial roles in various biological processes such as energy metabolism (e.g., glucose), precursor for inflammatory signaling (e.g., arachidonic acid), and neurotransmission system (glycine and valine).

Notably, compared to the control group, the fentanyl overdose group showed a significant reduction in carbohydrates glucose and turanose levels (*p* < 0.01) (Figure 5). The depletion in the level of carbohydrates indicates that there is an excessive need for energy production and an increase in brain activities. Neither ceftriaxone nor MC-100093 was able to fully restore the metabolites levels to the same level as the control group (Figure 5). The levels of both glucose and turanose in both β-lactams groups remained significantly lower than the control (*p* < 0.01).

For amino acids, the fentanyl overdose group showed a significant reduction in glycine (*p* < 0.05) and *L*-valine (*p* < 0.0001) levels compared to the control group (Figure 6), which may indicate a disruption in the neurotransmission system of the reward system. Depletion of glycine, which is inhibitory neurotransmitter, points to increase brain activities, while depletion of valine supports a dysregulation in the Glutamine–Glutamate–GABA axis.

For fentanyl overdose-induced valine depletion, neither ceftriaxone nor MC-100093 was able to fully restore the metabolites levels to the same level as the control group. However, both β-lactams were able to prevent the significant reduction in the level of glycine that was observed in the fentanyl overdose group. The metabolite levels in the fentanyl overdose, ceftriaxone/fentanyl overdose, and MC-100093/fentanyl overdose groups were not significantly different from the control group, suggesting that both treatments were effective in counteracting the effects of the fentanyl overdose on the glycine level.

Moreover, the drug overdose significantly decreased fatty acids arachidonic acid (*p* < 0.05) and mono palmitin (*p* < 0.01) levels compared to the control group (Figure 7). Depletion of fatty acids arachidonate, which is a precursor for inflammatory mediators and highly expressed in cell membranes, may indicate cell membrane damages and synthesizing signaling molecules for inflammation in the NAc.

For fentanyl overdose-induced palmitin depletion, neither ceftriaxone nor MC-100093 was able to restore the palmitin level. For the arachidonate level, ceftriaxone did not restore fentanyl overdose-induced depletion to the control group’s levels. In contrast, MC-100093 was able to prevent the significant reduction in arachidonate levels caused by the fentanyl overdose. The arachidonate levels in the fentanyl overdose and MC-100093 group were not significantly different from the control group, suggesting that MC-100093 was effective in counteracting the effects of the fentanyl overdose on arachidonic acid.

**Figure 8:**
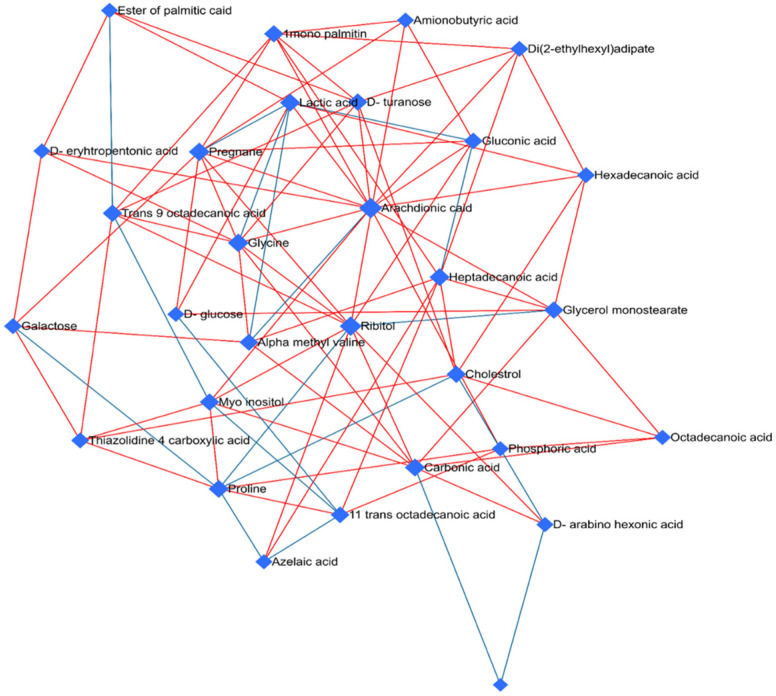
Network analysis of disrupted metabolites in the fentanyl overdose group. Network analysis illustrating the interactions and relationships between significantly altered metabolites in the fentanyl overdose group. Each node represents a specific metabolite, while edges (lines) connecting the nodes indicate known biochemical interactions. The size of each node corresponds to the magnitude of change in metabolite level, with larger nodes representing greater alterations. The color of the edges represents the type of correlation between metabolites: red edges denote positive correlations (indicating that as the level of one metabolite increases, the level of the connected metabolite also increases), while blue edges indicate negative correlations (suggesting that as the level of one metabolite increases, the level of the connected metabolite decreases). These correlations were determined based on the Pearson correlation coefficients derived from the metabolomic data. Central hub metabolites, such as arachidonic acid, ribitol, and glycine, are prominently affected, indicating their critical role in the response to fentanyl toxicity. The network highlights the complexity of metabolic alterations and the interconnected nature of disrupted pathways, providing insights into the biochemical impact of fentanyl overdose. This analysis underscores the extensive metabolic reprogramming that occurs in response to fentanyl toxicity.

Furthermore, we conducted a network analysis to evaluate the relationships between metabolites, understand metabolic pathways, and uncover system-level changes associated with fentanyl overdose (Figure 8). A correlation-based network was constructed, where nodes represented metabolites and edges represented statistically significant correlations (*p* < 0.05) between metabolite pairs. Nodes with high centrality measures may be considered as key metabolites within the network. The network structure suggested potential regulatory relationships between metabolites. For instance, arachidonic acid (13), ribitol (11), glycine (9), pregnane (9), carbonic acid (8), heptadecanoic acid (8), proline (8), and lactic acid (8) showed a high degree of centrality and were linked to several other metabolites, implying a central regulatory role in the metabolic network.

**Figure 9:**
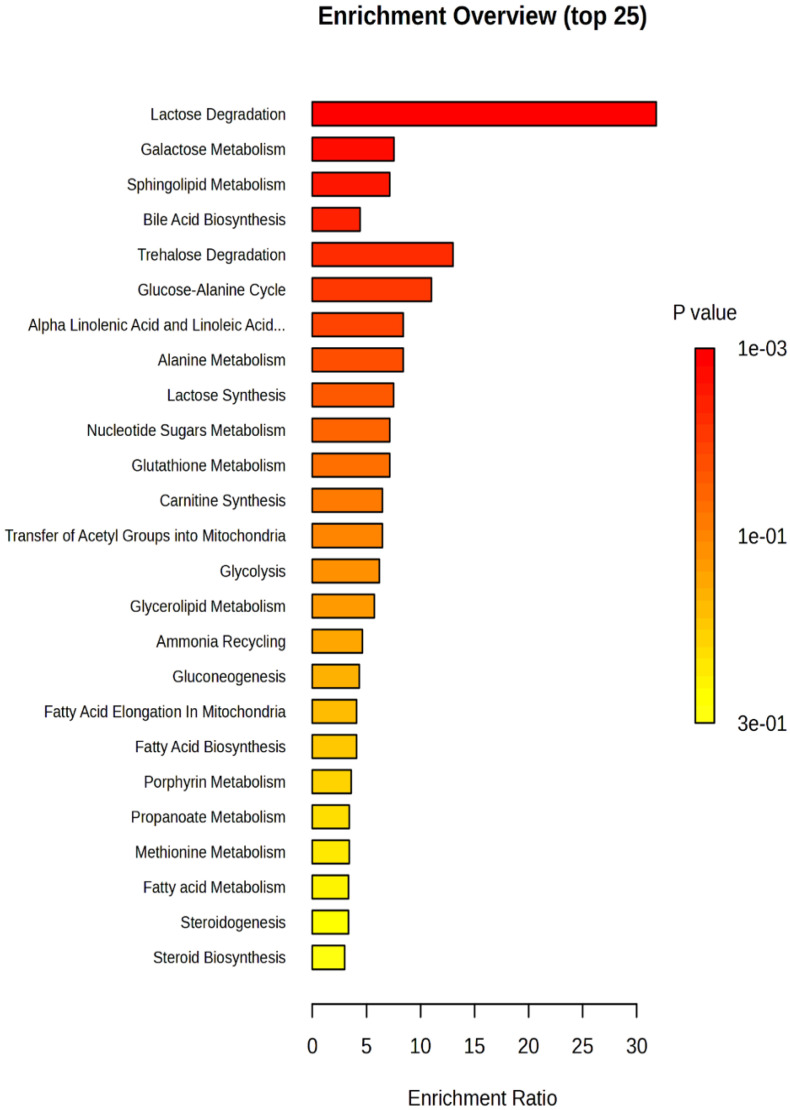
Enrichment analysis highlighting the significantly altered metabolic pathways in the fentanyl overdose group. The analysis was performed to identify which metabolic pathways were significantly impacted due to the fentanyl overdose. Each bar represents a different metabolic pathway, with the length of the bar corresponding to the degree of enrichment, measured by the enrichment score. The color of the bars indicates the statistical significance of the enrichment, with darker colors representing lower *p*-values (higher significance). Pathways shown include those involved in energy metabolism, lipid metabolism, and amino acid metabolism, inflammation processes, and others. The x-axis denotes the enrichment score, while the y-axis lists the specific metabolic pathways. Notably, pathways such as lactose/galactose metabolism, sphingolipid metabolism, and bile acid biosynthesis are significantly enriched, indicating a strong metabolic response to fentanyl toxicity.

In order to provide insights into the biological relevance and functional interpretation of metabolomic data, enrichment analysis was performed with associated differentially regulated metabolites as a result of fentanyl overdose with their metabolic pathways and biochemical processes (Figure 9). Pathway enrichment analysis revealed some metabolic pathways that were significantly enriched between the differentially regulated metabolites (*p* < 0.05). The top enriched pathways included lactose degradation, galactose metabolism, sphingolipid metabolism, and bile acid biosynthesis. Metabolites linked to energy metabolism, such as those involved in lactose degradation, and galactose metabolism exhibited significant alterations. This suggests alterations in energy production and utilization in case of exposure to a high dose of fentanyl. In addition, the enrichment analysis highlighted perturbations in lipid metabolism pathways, particularly sphingolipid metabolism and alpha linolenic acid metabolism. Also, an amino acid metabolism pathway, such as alanine metabolism, was affected. Moreover, alteration was detected in the glutathione metabolism, which is involved in detoxification, protection against oxidative stress, and maintenance of cellular redox balance. Importantly, glutamate is an essential component of glutathione, which may partially explain the beneficial effect of β-lactams such as ceftriaxone and MC-100093 as GLT-1 modulators in substance use disorders, including opioid use disorders. Metabolic pathway associated with glutamate was not statistically affected.

**Figure 10:**
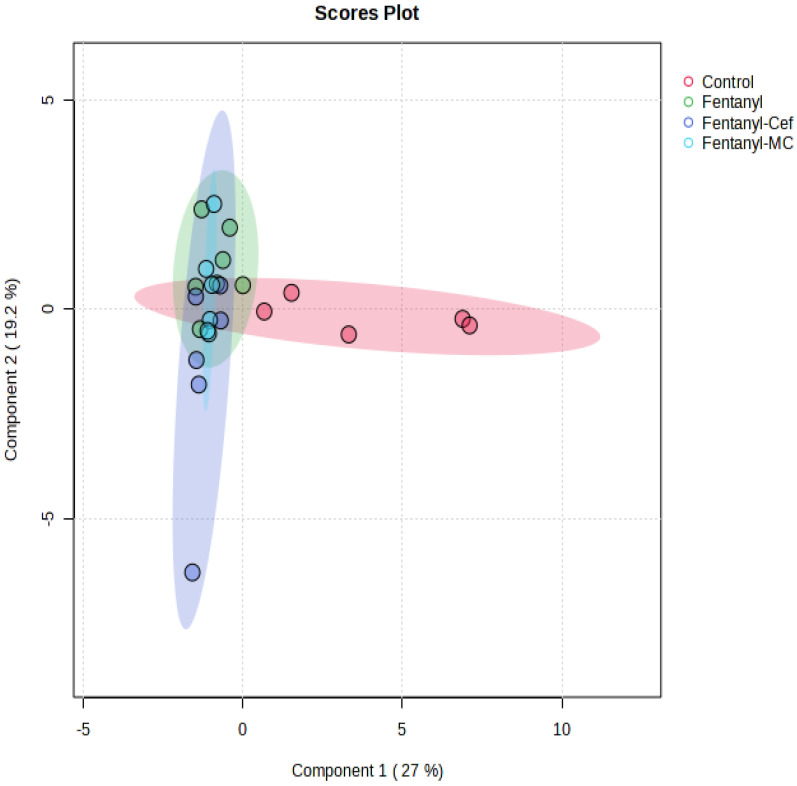
Sparse Partial Least Squares Discriminant Analysis. sPLS-DA score plot displaying the separation of metabolite profiles between the control group and fentanyl overdose groups. Red circles indicate control group samples, green circles fentanyl overdose group samples, purple circles fentanyl/ceftriaxone group samples, and light blue circles fentanyl/MC-100093 group samples. The distinct clustering of samples from the control and fentanyl overdose groups highlights the substantial metabolic differences induced by fentanyl toxicity. There is no clear separation between the fentanyl-alone group, and both fentanyl/β-lactams groups (n = 5–7/group).

We performed sPLS-DA to visually discriminate the effect of fentanyl overdose based on metabolic profiles (Figure 10). In addition, we assessed the metabolic changes associated with β-lactams treatment and predict their response based on metabolomic profiles. The score plot depicted a clear separation between the control and fentanyl groups in the reduced-dimensional space. The fentanyl and control groups showed distinct clusters, indicating a robust separation by the model. Despite some separation between the fentanyl alone and fentanyl/ceftriaxone groups, no clear clusters were formed, indicating non-significant effects of β-lactams at the level of metabolites.

### 3.4. Assessment of the Expression of GLT-1 and TLR4, and IL-6 in the Nucleus Accumbens

Target proteins of interest were examined using Western blot, including GLT-1 (known to be associated with synaptic plasticity and transmission) [25], TLR-4, and IL-6 (known to be associated with neuroinflammation) [26,27]. This study included the control, fentanyl alone, fentanyl/ceftriaxone, and fentanyl/MC-100093 groups to compare GLT-1 expression profiles that can be used partially as a surrogate biomarker for the improvement in the behavioral symptoms and neuroinflammation associated with fentanyl overdose.

**Figure 11:**
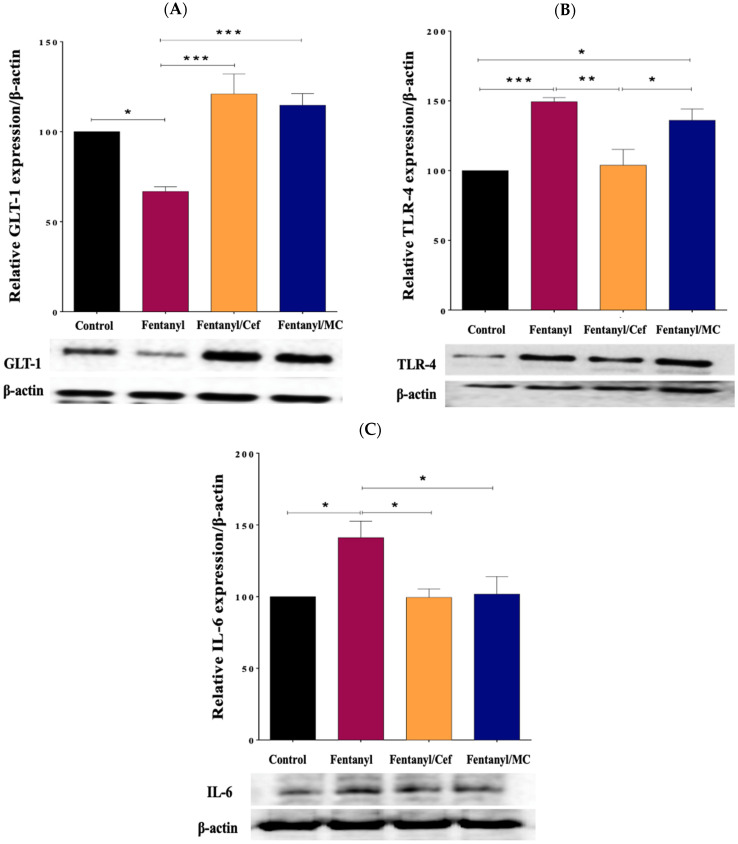
Effect of ceftriaxone and MC-100093 on the expression of GLT-1 (**A**), TLR-4 (**B**), and IL-6 (**C**) in the nucleus accumbens of the mouse model of fentanyl overdose (* *p* < 0.05, ** *p* < 0.01, *** *p* < 0.001, n = 5/group). Cef: ceftriaxone, MC: MC-100093, GLT-1: glutamate transporter 1, IL-6: interleukin 6, TLR-4: toll-like receptor-4.

GLT-1 protein bands were quantified, and the intensity of each band was normalized to β-actin. There were differential expression patterns of the target proteins between groups. Notably, GLT-1 showed a significant decrease in expression in the NAc of the fentanyl-alone group compared to the control (*p* < 0.01), and an increase in expression in both fentanyl/ceftriaxone (*p* < 0.001) and fentanyl/MC-100093 (*p* < 0.001) groups compared to the fentanyl-alone group as shown in Figure 11A.

In addition, the Western blot analysis revealed an improvement in inflammatory mediators, including TLR-4 and IL-6, corresponding to GLT-1 upregulation in ceftriaxone- and the MC-100093-treated groups compared to the fentanyl-alone group as shown in Figure 11B and C. One-way ANOVA followed by Tukey’s post hoc analysis was performed to assess the statistical significance of the observed differences in protein expression. With the exception of TLR-4 in fentanyl-exposed group versus fentanyl-MC-100093 group, the *p*-values associated with all proteins (control vs. fentanyl group, fentanyl vs. fentanyl-ceftriaxone group, and fentanyl vs. fentanyl-MC-100093 group) were below the significance threshold (e.g., *p* < 0.05), indicating reliable changes.

## 4. Discussion

In the current study, we identified for the first time a metabolomic profile associated with fentanyl overdose in the NAc of a mouse model. In addition, we evaluated the effect of ceftriaxone and MC-100093, known to upregulate GLT-1 expression, on behavioral manifestations and neuroinflammation associated with fentanyl overdose. We revealed that fentanyl overdose-induced hyperlocomotion and neuroinflammation were associated with a significant decrease in GLT-1 expression in the NAc. Although, ceftriaxone and MC-100093 did not attenuate fentanyl-induced alteration of target metabolites, these beta-lactams were able to reduce neuroinflammation and alleviate the severity of motor behaviors induced by fentanyl overdose. These therapeutic effects align with previously reported results about the beneficial effect of GLT-1 upregulation in hyperglutamatergic-excitotoxicity pathological conditions [7,14,28]. The induction of locomotor activities after fentanyl overdose is an indicative of the neurobehavioral changes, which are in the line with previous observation of acute fentanyl exposure in mice [29], supporting the validity of the current animal model. Elevation of the TLR-4 and IL-6 expression suggests a potential link between neuroinflammation and the observed neuropathological and behavioral changes. The identified neuroinflammatory mediators highlight potential therapeutic targets to mitigate the neurotoxic effects of acute fentanyl exposure.

In preclinical studies, several treatment paradigms were applied to investigate different aspects of opioid related effects, including fentanyl [30]. In contrast to the continuous paradigm, animals in the intermittent exposure paradigms are exposed to a stimulus in a non-continuous manner, with alternating exposure and non-exposure periods [30,31,32]. Intermittent exposure more closely resembles real-world conditions in many situations [30,31,33]. This paradigm can provide insights into the flexibility and adaptability of behaviors in response to intermittent stimuli, shedding light on how organisms adjust to varying environments. In contrast to continuous exposure, which can lead to tolerance and diminishing in the response over time, intermittent exposure helps mitigate tolerance, allowing researchers to observe sustained responses when the stimulus is reintroduced. Cowan et al. (2015) reported that intermittent, non-consecutive dosing schedule of an opioid agonist for 5 days (every other day, on day 1, 3, and 5) did not result in behavioral tolerance, compared to five consecutive days where the tolerance was clearly developed [34]. Similarly, Gaulden et al. (2021) investigated high doses of fentanyl using alternating dosing schedule of no-drug/drug conditioning (on days 1, 3, 5, 7, and 9) to measure drug abuse-related responses, and this paradigm successfully induced acute locomotor activity and behavioral sensitization in the tested animals [32]. By providing rest days between exposures, the development of tolerance and the impact of repeated but intermittent opioid administration can be reduced. Therefore, we decided in our study to administer fentanyl in an intermittent, non-consecutive days schedule, as previously described [32,34]. MC-100093 and ceftriaxone treatments started on day 5 to allow mice for habituation to fentanyl dosing from days 1 to5. In addition, the initial dosing of fentanyl (days 1, 3, and 5) may have an effect in GLT-1 expression, and we expected that MC-100093 and ceftriaxone dosing would attenuate this effect that might be observed with the initial and the later (days 7 and 9) dosing of fentanyl.

Induction of hypoventilation was used previously as an indicator of fentanyl overdose in mice [35]. A wide range of fentanyl doses (0.0032–32 mg/kg, i.p.) was studied in the context of hypoventilation, and dose-dependent effect with ED_50_ of 0.96 to 1.02 mg/kg was determined [35]. Similar results were found in another study in which studies demonstrated fentanyl induced hypoventilation in a dose range of (0.05–1.35 mg/kg, i.p.) [36]. Studies found that 0.05 mg/kg i.p. of fentanyl did not significantly affect respiratory function (tidal volume and minute volume) compared to the baseline values. Moreover, in locomotor activity tests, three different dosages of fentanyl, including 0.1 mg/kg, 1 mg/kg, and 10 mg/kg were evaluated, and it was found that 1 mg/kg and 10 mg/kg elicited significant dose-dependent increases in locomotor activity [35,36].

Therefore, since we aimed to administer an overdose that may pose a risk to animals, we decided to habituate the mice with intermittent repeated doses of 0.05 mg/kg, i.p., and then administered 1 mg/kg, i.p. as an overdose as recommended so that hypoventilation is modeled by a single function with an ED_50_ of 1 mg/kg [35,36]. We followed this paradigm with these carefully selected dosing schedules (1) to habituate animals and prepared them physiologically to receive the high dose, (2) to reduce the development of tolerance by providing rest days between exposures, (3) and to reflect real-world usage patterns as much as possible.

Our findings from the current study contribute to the understanding of the complex interactions between neurobiological changes and the behavioral phenotype of fentanyl overdose. We found that exposing mice to a high dose of fentanyl profoundly alters the metabolomic profiles in the NAc to an extent that indicates an unrecognized toxicological mechanism. The metabolomic profiling (Figure 3, Figure 7, Figure 8, Figure 9 and Figure 10) shows patterns of metabolite abundance upon exposure to a high dose fentanyl, providing clues about the perturbations in specific metabolic pathways. The heatmap analysis revealed distinct metabolic signatures associated with fentanyl overdose, suggesting potential biomarkers, and signaling pathways of interest for further investigation. Specific metabolites were identified with differential abundance between the control and fentanyl overdose groups (Figure 3, Figure 4, Figure 5 and Figure 6), which may serve as potential biomarkers and may offer insights into the metabolic alterations associated with fentanyl overdose.

The decrease in the abundance of amino acid-glycine, which is an inhibitory neurotransmitter [37], may suggest an increase in brain activity induced by fentanyl overdose [38]. Moreover, metabolism of the amino acid-alanine, which is also an inhibitory amino acid, was found to be disturbed, signifying the effect of fentanyl overdose on the excitability of the brain. Other amino acids were detected to be dysregulated, including valine and proline, which contribute to glutamate-glutamine metabolism, and an alteration in GABA pathway was observed to be correlated with these amino acids [39]. These findings were in line with previously published finding on opioids metabolomics, which found glycine, proline, and valine markedly disturbed [40,41]. Furthermore, metabolomic profile of methamphetamine-exposed animals was characterized previously, and it was noted that there was a reduction in the levels of glycine, valine, alanine, and methionine, consistent with our present findings [42,43].

We also found that an abundance of carbohydrate metabolites, such as glucose, galactose, and turanose, was affected in the brains of mice exposed to fentanyl overdose, which is a characteristic feature of metabolomic profiles of several drugs of abuse [38]. Carbohydrates play crucial roles in energy metabolism, glycosylation processes, and various cellular functions. Glucose is a central player in cellular energy metabolism, being a primary source of energy through glycolysis. Perturbations in glucose levels may reflect changes in energy demands or alterations in energy metabolic pathways. We recognized that the level of glucose metabolite was reduced significantly in brain tissue after fentanyl overdose; similar to what was found in the thalamus and striatum of rats exposed acutely to cocaine [44]. This depletion indicates an excessive need for energy production. Additionally, ribitol, which is a pentose alcohol involved in the pentose phosphate pathway, was found to be dysregulated in fentanyl overdose. It is a critical substrate in several dehydrogenase enzymes [45], and it was identified as one of the metabolites associated with Alzheimer’s disease [46]. Moreover, myo-inositol, which is the most abundant metabolite in the brain, mainly in glial cells [47], was also found to be significantly decreased as a result of fentanyl overdose. It is critically involved in several metabolic pathways, including inositol phosphate metabolism, and it has been allocated as a biomarker associated with Alzheimer’s disease and schizophrenia [46,48]. In line with the present finding, the abundance of myo-inositol was altered after exposure to morphine [49,50], cocaine [51], and methamphetamine [43], indicating a significant impact on the glial cell’s function.

Importantly, we noted an increase in the abundance of lactic acid, which plays a role in several metabolic pathways, including pyruvate metabolism, Warburg effect, and gluconeogenesis. Lactate is a part of the adaptive response when the oxygen supply does not match the physiological needs after energy failure occurs and transfer to anaerobic metabolism. The level of lactic acid is used to assess the severity of energy depletion as a result of respiratory failure. Increased level of lactate may be indicative of respiratory depression, which is associated with nerve damage [52]. An increased level of lactate metabolite is correlated with mitochondrial dysfunction and identified as a biomarker by metabolomic profiling of Parkinson’s disease [53], acute methamphetamine exposure [42,54,55], and cocaine abuse [56].

Regarding lipid metabolism, the metabolomics profiling of fentanyl overdose identifies specific lipids that show changes in abundance. This includes various classes of lipids such as phospholipids, glycerolipids, and sphingolipids. Generally, lipid-related compounds are involved in cell membrane stability and energy utilization. Glycerol monostearate, commonly known as monoacylglycerol, is an organic molecule that belongs to the glycerol ester family, and it is synthesized through the esterification of glycerol and stearic acid, which is a long-chain fatty acid [45]. Monoacylglycerol is involved in several biological processes, including lipid peroxidation, fatty acid metabolism, cell signaling, and lipid metabolism pathway [57]. Its abundance has been highlighted to increase with fentanyl overdose compared with the control group. Alternatively, levels of other lipids such as cholesterol, arachidonic acid, heptadecanoic acid, and monopalmitin were found to decrease with fentanyl overdose, implying that there was an increase in energy production or that they were consumed for synthesizing inflammatory mediators [58,59]. These findings are consistent with previous studies implicating dysregulation of lipid metabolism upon exposure to methamphetamine and heroin [43,60,61]. Importantly, arachidonic acid and heptadecanoic acid were identified as a prospective biomarker for schizophrenic individuals [62,63], explaining partially the phenotypic similarities between fentanyl overdose and schizophrenia.

To gain insights into the underlying biological pathways and functions associated with the observed changes in metabolite profiles, we performed enrichment analysis on the metabolomic dataset (Figure 9). Our analysis revealed three enriched pathways that are significantly associated with the altered metabolites. Notably, the top three enriched pathways, lactose degradation, galactose metabolism, and sphingolipid metabolism, suggest a coordinated response in these metabolic pathways in fentanyl overdose. The lactose degradation pathway involves the breakdown of lactose into glucose and galactose. The enrichment of this pathway illustrates that there is a need for more glucose to feed the TCA cycle for energy production. Consistently, metabolic fate of these carbohydrates, including glycolysis and gluconeogenesis, was enriched, implying the overall dysregulation in the energy metabolism and utilization, including the TCA cycle. Future metabolomics studies are warranted to investigate the levels of enzymes involved in lactose degradation, such as lactase, and enzymes downstream in the metabolic pathways of glucose and galactose. This includes enzymes like hexokinase, which phosphorylates glucose, and galactokinase, which phosphorylates galactose. Enrichment of pathways related to lipid metabolism, particularly sphingolipid metabolism, bile acid biosynthesis, linoleic acid metabolism, fatty acid metabolism, carnitine synthesis, and glycerolipid metabolism, suggests a pronounced impact on cellular energy balance and neuroinflammatory response to fentanyl overdose [64]. Carnitine is crucial for the transport of long-chain fatty acids into the mitochondria for β-oxidation. The enrichment of carnitine synthesis indicates the elevation of breakdown of fatty acids through β-oxidation [65].

Regarding the effects of β-lactam on metabolomics of fentanyl overdose, generally there was no significant changes in the overall metabolites between the fentanyl alone and fentanyl β-lactams-treated groups. However, enrichment analysis revealed some changes in the glutamate metabolism pathway between the fentanyl-alone group and fentanyl/β-lactams-treated group. This suggests a potential involvement of this pathway in the overall therapeutic effect.

The observed changes in TLR-4 and IL-6 suggest potential alterations in inflammatory pathways in the case of fentanyl overdose. Consistently, the enrichment analysis revealed a significant enrichment in sphingolipids metabolism pathway, which is strongly correlated with neuroinflammation and has been identified recently as a diagnostic and therapeutic target [64]. The effects of β-lactams on GLT-1, TLR-4, and IL-6 were compared and validated with the existing literature on substance use disorders [7,14,28]. Consistency with previous findings strengthens the reliability of this study’s results. Even though ceftriaxone and MC-100093 upregulated GLT-1 significantly with subsequent alleviation in inflammatory biomarkers, the disrupted metabolites as a result of fentanyl overdose were not restored, indicating independent responses.

In this study, we exclusively utilized male mice, which may limit the generalizability of our findings to female. A recently published study showed that biological sex is a crucial determinant in drug dependence vulnerability and influences the mechanisms involved in opioid use, including fentanyl [66]. Notably, sex differences were observed in behavioral and physiological responses to fentanyl, including affective symptoms, withdrawal, tolerance, and analgesic effect. Given these differences, it is important to consider that female mice may exhibit distinct responses to fentanyl overdose compared to their male counterparts. Therefore, while our findings provide valuable insights into the male response to fentanyl, they should be interpreted with caution when extrapolating to females. Further studies incorporating female mice are essential to fully understand the sex-specific mechanisms of fentanyl overdose to draw more comprehensive and generalizable conclusions.

## 5. Conclusions

In summary, the exploration of the metabolic profile of the NAc in a fentanyl overdose mouse model provides valuable insights into the complex interplay between neurochemistry and the pathophysiology of opioid overdose. Our findings revealed alterations in key metabolites and metabolic pathways associated with neurotransmitter regulation, energy metabolism, and oxidative stress within the brains of mice subjected to fentanyl overdose. The identification of these neurochemical signatures not only contributes to our understanding of the underlying mechanisms but also holds promise for the development of novel diagnostic markers and targeted therapeutic interventions. Even though no significant metabolomics change, enhancing glutamate clearance by upregulation of GLT-1 expression mitigates the neuroinflammation and behavioral changes associated with fentanyl overdose, suggesting a promising approach for therapeutic intervention.

## Data Availability

The data presented in this study are available within the article.
